# Kinin Receptors B1 and B2 Mediate Breast Cancer Cell Migration and Invasion by Activating the FAK-Src Axis

**DOI:** 10.3390/ijms252111709

**Published:** 2024-10-31

**Authors:** Felipe González-Turén, Lorena Lobos-González, Alexander Riquelme-Herrera, Andrés Ibacache, Luis Meza Ulloa, Alexandra Droguett, Camila Alveal, Bastián Carrillo, Javiera Gutiérrez, Pamela Ehrenfeld, Areli Cárdenas-Oyarzo

**Affiliations:** 1 School of Nursing, Faculty of Health Sciences, Universidad Bernardo O’Higgins, Santiago 8370854, Chile; 2 Laboratory of Cellular Communication, Program of Cell and Molecular Biology, Biomedical Sciences Institute (ICBM), Faculty of Medicine, Universidad de Chile, Santiago 8370854, Chile; 3 Center for Regenerative Medicine, Institute for Sciences and Innovation in Medicine, Facultad de Medicina, Clínica Alemana Universidad del Desarrollo, Santiago 7610658, Chile; 4 Centro Integrativo de Biología y Química Aplicada (CIBQA), Faculty of Health Sciences, Universidad Bernardo O’Higgins, Santiago 8370854, Chile; 5 Laboratory of Molecular Design, Faculty of Biological Sciences, Pontificia Universidad Católica de Chile, Santiago 8370854, Chile; 6 School of Medical Technology, Faculty of Health Sciences, Universidad Bernardo O’Higgins, Santiago 8370854, Chile; 7 Laboratory of Cellular Pathology, Institute of Anatomy, Histology & Pathology, Faculty of Medicine, Universidad Austral de Chile, Valdivia 5110566, Chile; 8 Center for Interdisciplinary Studies on the Nervous System (CISNe), Universidad Austral de Chile, Valdivia 5110566, Chile; 9 School of Obstetrics and Puericulture, Faculty of Medical Sciences, Universidad Bernardo O’Higgins, Santiago 8370854, Chile

**Keywords:** kinin receptors, B1 receptor, B2 receptor, migration, invasion, breast cancer, FAK, Src

## Abstract

Kinin receptors B1 and B2 are involved in migration and invasion in gastric, glioma, and cervical cancer cells, among others. However, the role of kinin receptors in breast cancer cells has been poorly studied. We aimed to reveal the impact of B1 and B2 receptors on migration and invasion in breast cancer cells and demonstrate their capacity to modulate in vivo tumor growth. MDA-MB-231, MCF-7, and T47D cells treated with Lys-des[Arg^9^]bradykinin (LDBK) or bradykinin (BK) were used to evaluate migration and invasion. Des-[Arg^9^]-Leu^8^-BK and HOE-140 were used as antagonists for the B1 and B2 receptors. MDA-MB-231 cells incubated or not with antagonists were subcutaneously inoculated in BALBc NOD/SCID mice to evaluate tumor growth. LDBK and BK treatment significantly increased migration and invasion in breast cancer cells, effects that were negated when antagonists were used. The use of antagonists in vivo inhibited tumor growth. Moreover, the migration and invasion induced by kinins in breast cancer cells were inhibited when focal adhesion kinase (FAK) and Src inhibitors were used. The novelty revealed in our work is that B1 and B2 receptors activated by LDBK and BK induce migration and invasion in breast cancer cells via a mechanism that involves the FAK–Src signaling pathway, and the antagonism of both receptors in vivo impairs breast tumor growth.

## 1. Introduction

Breast cancer is a disease caused by the abnormal and disordered growth of epithelial cells in mammary ducts or lobules that can spread [1]. In women, breast cancer is the number one cause of cancer deaths worldwide, estimated at 626,000 deaths in 2020, with a standardized mortality rate of 6.9% and an incidence rate of 11.7% [2]. Most deaths (90%) in breast cancer patients are caused by the invasion and metastasis of the primary tumor [3,4], mainly because a high number of patients are diagnosed at the advanced stages of the disease [1,5]. Thus, novel biomarkers are required for the design of new strategies to treat this cancer, and kinin receptors could be a target.

Kinins, which are biologically active peptides, play a significant role in both physiology and pathophysiology. In physiological conditions, they are involved in regulating blood pressure, promoting vasodilation, and enhancing vascular permeability. They also contribute to pain sensation and inflammation, acting as mediators that help the body respond to injury [6]. In pathophysiological contexts, the overproduction or dysregulation of kinins can lead to various conditions, including chronic pain, allergic reactions, and inflammatory diseases [7,8]. Their involvement in processes such as tissue remodeling underscores their importance in both normal biological functions and disease states.

Cumulative evidence has also shown the involvement of kinin receptors in cell migration and invasion under inflammation and cancer conditions.

Kinin receptors are biological effectors of the kallikrein-related peptidase family. In mammals, including humans, these two classical kallikreins generate either the decapeptide kallidin (Lys-bradykinin) or the nonapeptide bradykinin (BK), the principal agonist of the kinin B2 receptor (BDKR2 gene, B2R). Once formed, and by proteolytic cleavage of the C-terminal arginine, these peptides may be converted into Lys-des[Arg^9^]bradykinin (LDBK) or des[Arg^9^]bradykinin (DBK), agonists of the kinin B1 receptor (BDKR1 gene, B1R) [7,8].

Nonapeptide BK can induce migration in glioma cells, an effect that is mediated by activating PI3K, AKT, c-Jun, and activator protein 1 (AP-1) signaling. BK also induces the invasion of glioma cells when acting as a chemoattractant, guiding the cells towards blood vessels [9,10]. On malignant glioblastoma cells, BK promotes migration by activating B1R and is mediated by the Ca^2+^-MEK1-ERK1/2-NF-kB pathway [10]. Similarly, BK induces the acquisition of the pro-metastatic phenotype of neuroblastoma cells, which is characterized by an increase in VEGF, the induction of MMP-2 expression, and the gelatinase activity of MMP-2 and MMP-9, keys for extracellular matrix degradation during invasion [11]. 

Accordingly, the activation of B1R on prostate cancer PC3 cells promotes migration mediated by the phosphorylation of focal adhesion kinase (FAK) [12]. Further, on prostate cells, BK, through B2R, enhances chemomigration and MMP-9 expression, effects mediated by PKCδ, c-Src, and NF-κB [13].

On gastric cancer cells, BK promotes proliferation, migration, and invasion. Furthermore, BK causes the up-regulation of p-ERK1/2 and MMP-2/MMP-9 and the down-regulation of E-cadherin [14]. BK, through B2R, also enhances cell proliferation, migration, and invasion in cervical cancer cells; all these effects are mediated by STAT3 signaling pathways [15].

On breast cancer cells MCF-7 and ZR-75-1 (estrogen-sensitive cells), the B1R agonist induces proliferation [16,17]. Moreover, kinins, through B1R, lead to the activation of ERK1/2 signaling pathways, the result of which is the promotion of MMP2 and MMP9 release, crucial for tumor invasion [17,18]. Despite these findings, the impact of B1R and B2R on migration and invasion in breast cancer cells is not fully understood. The role of the B2 receptor in breast cancer cell migration and adhesion is unknown. Therefore, our work aimed to evaluate the impact of B1R and B2R activation on migration and invasion, examine their associated mechanisms in breast cancer cells, and study the effect of the antagonism of both receptors in vivo.

The novelty revealed in our study is that B1 and B2 receptors activated by nonapeptides, LDBK, and BK induce migration and invasion in breast cancer cells via a mechanism that involves the FAK–Src axis, and the antagonism of both receptors in vivo inhibits breast tumor growth.

## 2. Results

### 2.1. Kinin Peptides Enhance the Migration Capacity of Breast Cancer Cells

Kinin receptors mediate migration and invasion in cancer cells. Our previously published data showed that LDBK, through the B1 receptor, increased the release of MMPs, which are implied in migration and extracellular matrix invasion. Because of this, we evaluated the impact of kinin nonapeptides LDBK and BK on migration and invasion in MDA-MB-231, MCF-7, and T47D cells. We assessed migration using scratch and transwell assays. Wounds were developed on MDA-MB-231 cells after 30 min of incubation with the anti-proliferative compound mitomycin C. 

**Figure 1:**
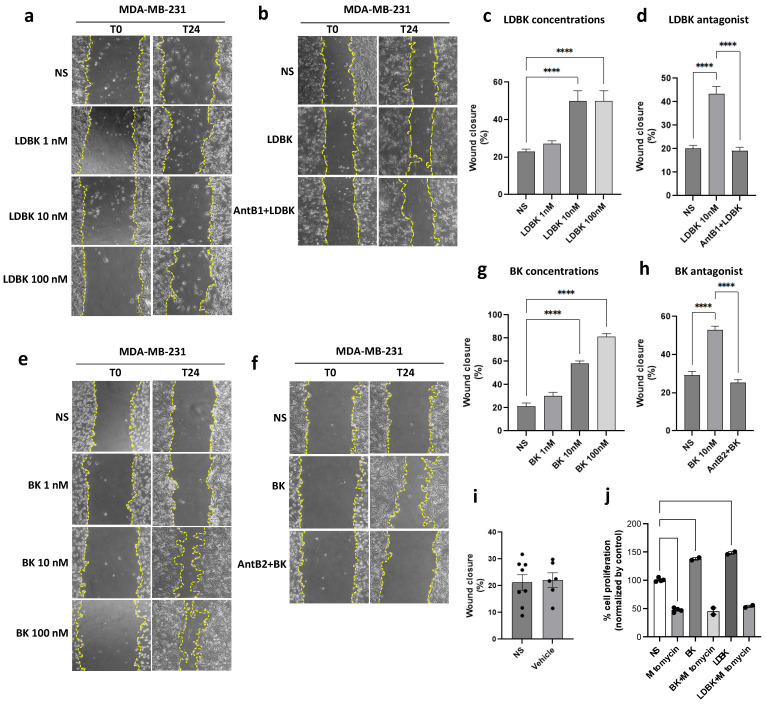
LDBK and BK, through B1 and B2 receptors, increase wound closure in MDA-MB-231 cells. (**a**,**e**): scratch assay in MDA-MB-231 cells treated with growing concentrations (1, 10, and 100 nM) of LDBK or BK; (**b**,**f**): the effect of B1 (AntB1) and B2 (AntB2) antagonists on wound closure induced by 10 nM of LDBK or 10 nM of BK, respectively (pictures magnification corresponds to 100×); (**c**,**d**,**g**,**h**): the graphs illustrate the closure percentage after 24 h of culture compared with non-stimulated cells (NS); (**i**) the graph exhibits the effect of the vehicle on wound closure in MDA-MB-231 cells after 24 h of scratch testing; and (**j**) the graph shows the effect of mitomycin C on the cell viability of MDA-MB-231 cells induced by LDBK and BK. Data are presented as the average ± standard error for at least three independent experiments. **** *p* < 0.0001. An ANOVA two-tailed test was used.

MDA-MB-231 cells treated for 24 h at increasing concentrations of LDBK (1, 10, and 100 nM) after scratch injury presented an increase in the wound healing percentage compared with the non-stimulated cells (Figure 1a,c). Moreover, the effect of LDBK was not dose-dependent, presenting no differences between 10 nM and 100 nM conditions. 

Conversely, BK also increased scar closure compared with the non-stimulated cells in a dose-dependent manner (Figure 1e,g). To assess the participation of B1 and B2 receptors, cells were treated with their antagonists. Cells were incubated with des-[Arg^9^]-Leu^8^-BK (AntB1) or HOE-140 (AntB2) for one hour prior to the addition of the agonists. Both antagonists effectively inhibited the effects of kinins, since the presence of des-[Arg^9^]-Leu^8^-BK and HOE-140 did not lead to an increase in the wound closure percentage, which remained similar to that of the non-stimulated cells, approximately 20% (Figure 1b,d,f,h). In addition, we investigated whether the vehicle used to resuspend the peptides affected the action of kinins on the cells. The results demonstrated that the vehicle did not impact this effect (Figure 1i). Additionally, scratch assays were performed in MCF7 cells, where the results were like those observed in MDA-MB-231 cells (Supplementary Material: Figure S1).

As a control, a cell viability assay was performed. Mitomycin C was used owing to the proliferative effect of kinins on breast cancer cells. To assess the anti-growing effect of mitomycin C on cells stimulated with kinins, an MTT assay was performed. The results demonstrated that mitomycin C inhibited the growing effect of LDBK and BK on the MDA-MB-231 cells (Figure 1j). To complement these results, a Trypan Blue dye exclusion assay was performed, where the results were consistent (Supplementary Material: Figure S2). Consequently, it was determined that the effect of kinins on wound closure is due to migration more than proliferation (Supplementary Material: Figure S3).

**Figure 2:**
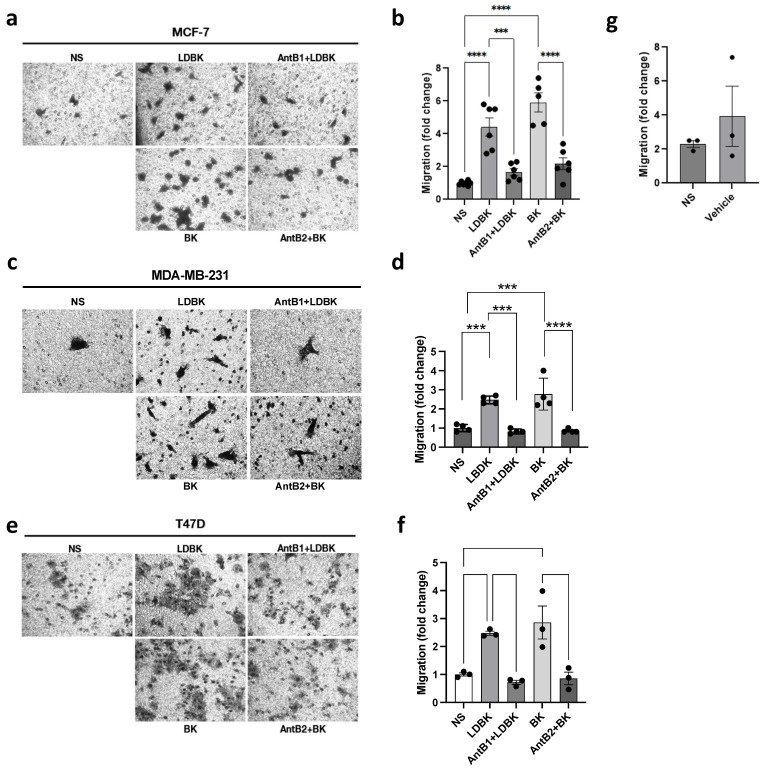
LDBK and BK, through B1 and B2 receptors, increase transwell migration in MCF-7, MDA-MB-231, and T47D cells. (**a**,**c**,**e**): the effects of LDBK and BK, and their antagonists, on transwell migration in MCF-7, MDA-MB-231, and T47D cells (pictures magnification corresponds to 400×); (**b**,**d**,**f**): the graphs show the fold change in stimulated migrating cells compared with the control. The number of migrating cells was normalized by the non-stimulated cells (NS), and (**g**) the graph shows the effect of the vehicle on the invasion of the MDA-MB-231 cells after 24 h of stimulus. Data are presented as the average ± standard error for at least three independent experiments. *** *p* < 0.001, and **** *p* < 0.0001. An ANOVA two-tailed test was used.

Transwell assays were developed to complement the results obtained using scratch assays. The cells were seeded in transwell inserts, and a B1 or B2 agonist was added. The results demonstrated that stimulation with 10 nM of LDBK or 10 nM of BK for 24 h increased the migration capacity of the MCF-7 and T47D cells (Figure 2a–d). We observed that the number of cells in the bottom of the inserts was greater under kinin-stimulated conditions compared with the non-stimulated cells. The contribution of the B1 and B2 receptors was assessed by adding their antagonists. In both the MCF-7 and T47D cells, des-[Arg^9^]-Leu^8^-BK (AntB1) and HOE-140 (AntB2) impaired the migration induced by LDBK and BK. Therefore, it was found that kinin nonapeptides can increase the migration capacity in breast cancer cells, and these effects are mediated by B1 and B2 receptors.

### 2.2. Kinin Peptides Improve the Invasion Capacity of Breast Cancer Cells

**Figure 3:**
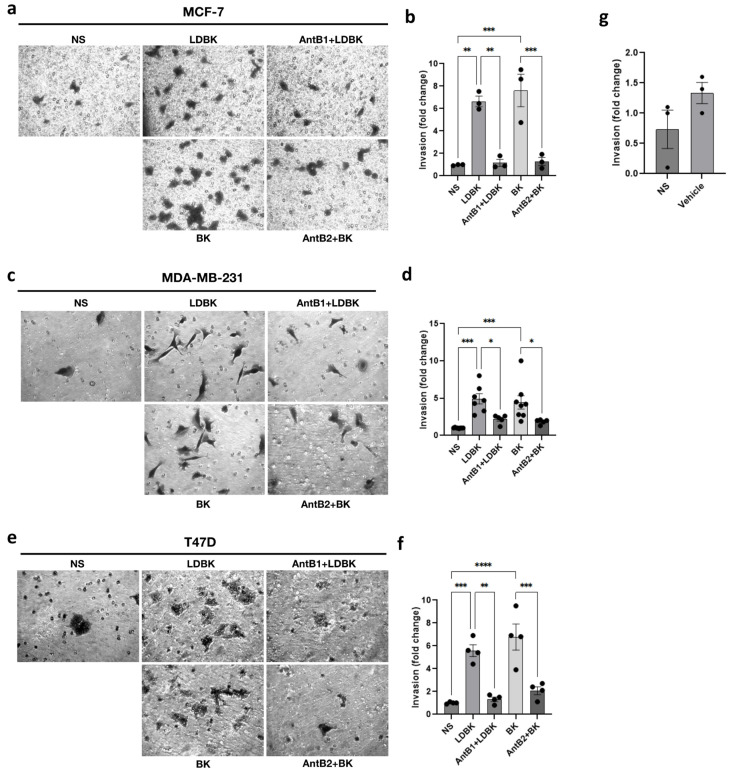
B1 and B2 receptors mediate the kinin-induced invasion of breast cancer cells. (**a**,**c**,**e**): the effects of LDBK and BK, and their antagonists, on Matrigel invasion in MCF-7, MDA-MB-231, and T47D cells (pictures magnification corresponds to 400×); (**b**,**d**,**f**): the graphs show the fold change in stimulated invasive cells compared with the control. The number of invasive cells was normalized by the non-stimulated cells (NS), and (**g**) the graph shows the effect of the vehicle on the invasion of MDA-MB-231 cells after 24 h of stimulus. Data are presented as the average ± standard error for at least three independent experiments. * *p* < 0.05, ** *p* < 0.01, *** *p* < 0.001, and **** *p* < 0.0001. An ANOVA two-tailed test was used.

Cancer cells are able to migrate and are capable of invading the extracellular matrix, a step prior to and relevant for the metastasis step. For this reason, we studied the impact of LDBK and BK on Matrigel invasion in MDA-MB-231, T47D, and MCF-7 cells (Figure 3a,c,e). Elevated numbers of cells were capable of passing through Matrigel after 24 h of treatment with B1 and B2 agonists compared with the non-stimulated cells (Figure 3b,d,f). The effects of the antagonists were opposite to those of the agonists. Therefore, invasion, similar to migration, was enhanced by LDBK and BK, and treatment with their antagonists des-[Arg^9^]-Leu^8^-BK (AntB1) and HOE-140 (AntB2) had the opposite effect, demonstrating the participation of B1 and B2 receptors in these processes.

### 2.3. Kinin Peptides Enhance the Migration and Invasion Capacity of Breast Cancer Cells Through Src- and FAK-Dependent Pathways

**Figure 4:**
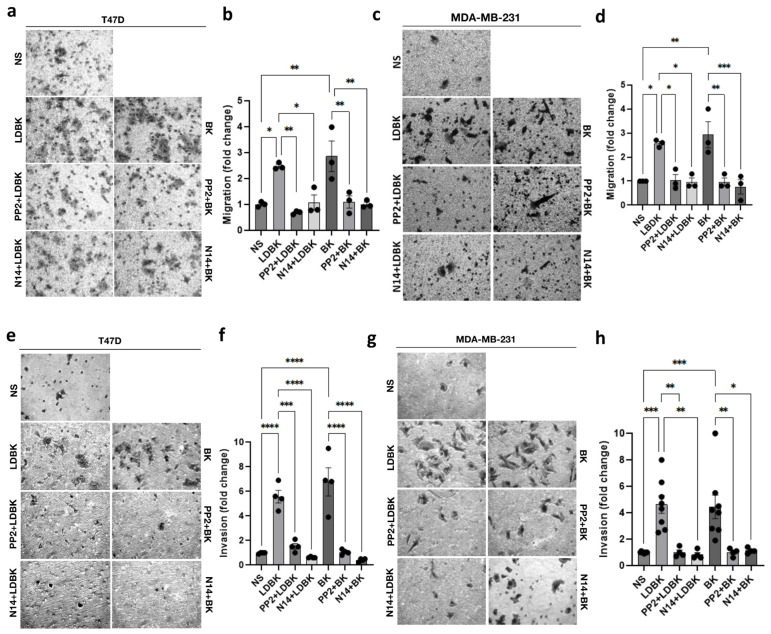
Migration and invasion induced by kinin nonapeptides in breast cancer cells are mediated by Src and FAK. (**a**,**c**): transwell assay of T47D and MDA-MB-231 cells incubated with Src (PP2) and FAK (N14) inhibitors before the addition of kinins. Cells non-stimulated with agonists or inhibitors were considered controls (NS); (**b**,**d**): the graphs show the fold change in the stimulated T47D and MDA-MB-231 migrating cells compared with the non-stimulated cells. The number of migrating cells was normalized by the control cells; (**e**,**g**): Matrigel assay of T47D and MDA-MB-231 cells incubated with PP2 and N14 inhibitors plus LDBK or BK for 24 h. Cells without stimuli were considered controls (NS); and (**f**,**h**): the graphs show the fold change in the stimulated T47D and MDA-MB-231 invasive cells compared with the non-stimulated cells. The number of invasive cells obtained in each condition was normalized by the control cells. Pictures magnification corresponds to 400×. Data are presented as the average ± standard error for at least three independent experiments. * *p* < 0.05, ** *p* < 0.01, *** *p* < 0.001, and **** *p* < 0.0001. An ANOVA two-tailed test was used.

FAK and Src are kinases implied in cell adhesion and migration. To assess this, we used specific inhibitors for Src (PP2) and FAK (N14). MDA-MB-231 and T47D cells were treated with inhibitors plus LDBK or BK as appropriate. Both inhibitors, PP2 and N14, were capable of impeding the increase in migration induced by LDBK and BK in the MDA-MB-231 and T47D cells (Figure 4a–d). The levels of migration were similar to those in non-stimulated cells. Moreover, inhibitors for FAK and Src also impaired the invasion promoted by kinin nonapeptides (Figure 4e–h). Treatment with FAK and Src inhibitors reverses the effect of kinins, acting as a control condition. In addition, the effects of inhibitors on migration and invasion were also tested in MCF7 cells; results were similar to those observed in T47D and MDA-MB-231 cells (Supplementary Material: Figures S4 and S5).

Therefore, the increase in migration and invasion induced by LDBK and BK involves FAK and Src activation signaling.

### 2.4. B1 and B2 Antagonists Prevent Tumor Growth Induced by MDA-MB-231 Cells Inoculated Subcutaneously in BALBc NOD/SCID Mice

**Figure 5:**
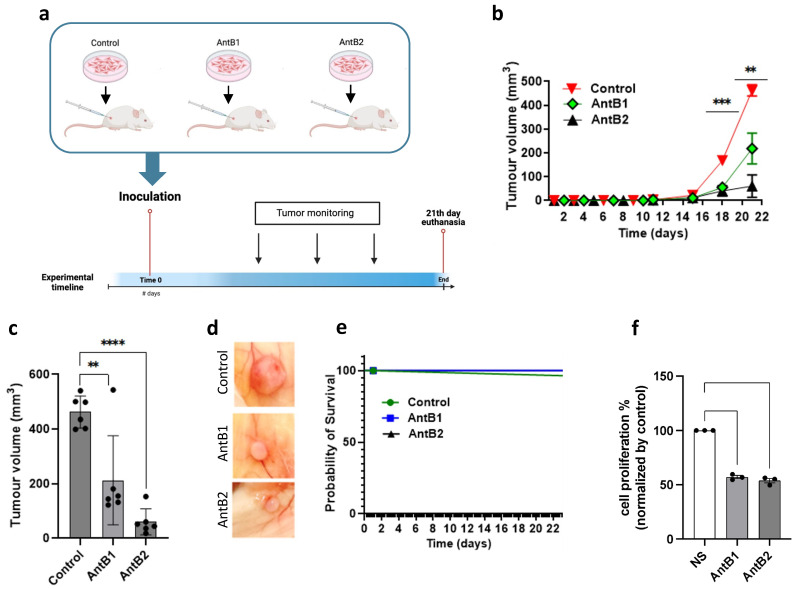
B1 and B2 antagonists impair tumor growth in vivo: (**a**) diagram of the protocol for breast cancer tumor formation based on the subcutaneous inoculation of MDA-MB-231 cells. Previously inoculated cells were treated for 24 h with B1 (AntB1) or B2 (AntB2) antagonists. Non-stimulated cells were used as controls; (**b**) tumor growth progression from the beginning to the 21st day; (**c**) tumor size at the end of the experiment (21st day); (**d**) representative pictures of tumor volume at different experimental conditions; (**e**) probability of the survival of mice under different conditions for 21 days; and (**f**) capacity of the proliferation of cells previously treated for 24 h with B1 and B2 antagonists. Data of six animals per group are presented as the average ± standard deviation. ** *p* < 0.01, *** *p* < 0.001, and **** *p* < 0.0001. A *t*-test was used.

To assess the effect of B1 and B2 antagonists in vivo, MDA-MB-321 cells treated or not with antagonists were inoculated (Figure 5a). Approximately 24 h before inoculation, the cells were incubated with antagonists B1 or B2 at a concentration of 1 µM. Once the cells were inoculated subcutaneously, tumor size was monitored for 21 days. After this, the mice were euthanized, and tumors were extracted and weighed. The tumor volume curve showed that the tumor started growing on the 15th day and that size differences, compared with saline-inoculated mice, were detected from the 18th day onward (Figure 5b,c). At the end of the experiment, the lump mass was found to be smaller in the antagonist-treated animals compared with the saline-treated animals (Figure 5d). The survival of the mice was not affected by the antagonists compared with the controls (Figure 5e). As a control, the effect of the antagonists on proliferation was evaluated at 24 h, which was the time the MDA-MB-231 cells were incubated with antagonists before inoculation. The results showed that the antagonists were able to inhibit the proliferation rate at 24 h. Therefore, when the cells were inoculated, their rate of proliferation diminished. Consequently, the pharmacological blocking of B1 and B2 receptors was found to inhibit tumor growth induced by MDA-MB-231 cells inoculated subcutaneously in female mice.

## 3. Discussion

**Figure 6:**
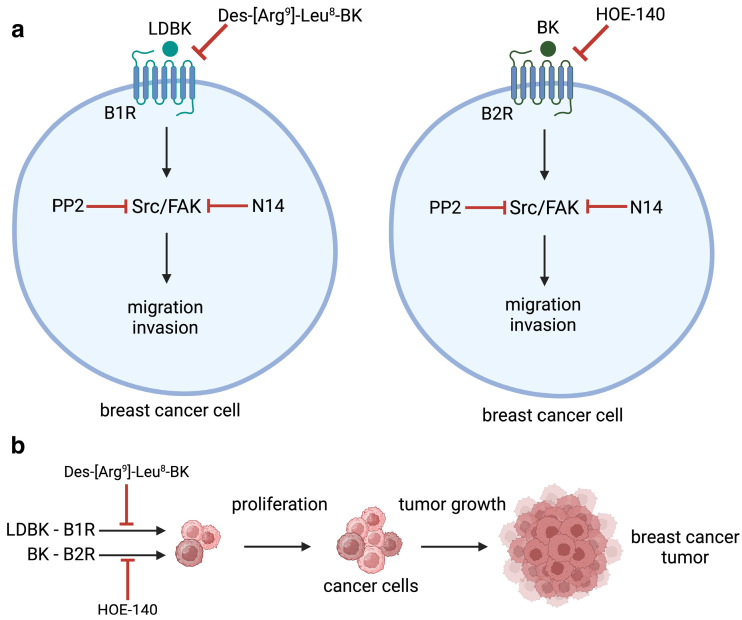
The role of kinin receptors in breast cancer cell migration, invasion, and tumor growth in vivo. This figure illustrates the proposed mechanism by which B1 and B2 receptor activation leads to enhanced cell motility and invasiveness, while receptor antagonism hinders tumor progression: (**a**) activation of B1 and B2 receptors by LDBK and BK induces migration and invasion in breast cancer cells through the FAK–Src signaling pathway; and (**b**) in vivo antagonism of both B1 and B2 receptors impairs breast tumor growth, highlighting the therapeutic potential of receptor blockade in breast cancer treatment.

The novelty revealed in our study is that B1 and B2 receptors activated by nonapeptides, LDBK, and BK induce migration and invasion in breast cancer cells via a mechanism that involves the FAK–Src axis (Figure 6a). 

Our experimental approach utilized MDA-MB-231, MCF-7, and T47D cell lines owing to their distinct biological characteristics, which are crucial for studying migration and invasion in breast cancer. MDA-MB-231 represents aggressive triple-negative breast cancer, serving as a model for exploring new therapies. In contrast, MCF-7 and T47D, both estrogen receptor-positive, facilitate the investigation of hormonal therapy effects on tumor progression. This combination enhances our understanding of metastatic mechanisms and informs the development of personalized treatment strategies, providing direct clinical value for breast cancer patients.

In breast cancer, B1 receptor expression has been detected on neoplastic tissue from both benign and malignant tumors. Similarly, breast cancer cell lines also express the B1 receptor [17,18,19]. Furthermore, both agonists, BK and DBK, have been found at elevated levels in the serum of breast cancer patients [20]. Notably, these levels decline following tumor removal, which parallels the findings in patients with cervical cancer [21].

Kinins are the products of the cleavage of kininogens of high or low molecular weight, which are expressed in breast cancer cell lines such as MCF-7, ZR-75, T47D, and MDA-MB-231. Moreover, the kinin-generating enzyme KLK1, responsible for cleaving kininogens, is also expressed in these cell lines, suggesting the local regulation of kallikrein-kinin production. The B1 receptor, activated by LDBK in MCF-7 cells, enhances the secretion of KLK1 and KLK6, where KLK1 produces ore kinins and KLK6 cleaves extracellular matrix proteins [22]. These findings suggest that kinins create a microenvironment that promotes tumor growth, migration, and invasion. Kinin receptors are also expressed in other cancer cells, where they modulate tumor proliferation, migration, and invasion.

According to our results, LDBK and BK stimulate migration, and this was corroborated by wound healing and transwell assays in MDA-MB-231 cells. Cells stimulated with LDBK or BK presented less closure area, demonstrating migration in the cells. To confirm that the closure was produced by migration, we used mitomycin C to inhibit proliferation, owing to previous findings that have shown that gastric, cervical, and breast cancer cells proliferate in response to kinin nonapeptides [14,15,16,17]. Mitomycin C effectively abolished proliferation, even when the cells were treated with LDBK and BK. Therefore, the effect that we observed in wound closure was due to cell migration. The use of antagonists revealed that the effects of LDBK and BK nonapeptides were mediated by the B1 and B2 receptors, respectively. In addition, we confirmed these results using transwell assays, in which we observed that B1 and B2 receptors modulated migration in estrogen-sensitive MCF-7 and T47D cells and highly metastatic MDA-MB-231 cells. Thus, it was found that both receptors regulate migration in estrogen-sensitive or non-estrogen-sensitive cells.

As mentioned above, there is evidence that kinins enhance the proliferation of cancer cells. It has been previously described that activation of the B1 and B2 receptors by their agonists LDBK and BK induces proliferation in estrogen-sensitive breast cancer cells (MCF7 and ZR-75-1 cells) and primary culture of breast cancer cells, which is mediated by the activation of ERK1/2 and is dependent on EGFR transactivation [16,17]. In agreement with our results, LDBK and BK were found to enhance the proliferation of MDA-MB-231 cells in vitro, and the use of B1 and B2 antagonists inhibited these effects in vivo (Figure 6b). 

Our findings are consistent with those previously published by Sun D-P et al., in which BK, through the B1 receptor, induced migration in malignant glioblastoma cells. The mechanism through which BK induced migration in these cells involved aquaporin-4 expression, activation of the MEK1–ERK1/2 signaling pathway, and the translocation and transactivation of NF-κB [10]. Another mechanism by which BK regulates migration in glioblastoma cells is the activation of STAT3 and the promotion of its interaction with SP-1 [23]. In cell lines from chondrosarcoma, BK also enhances migration, mediated by the activation of B1 and B2 receptors. The mechanism was found to involve the expression of α2β1 integrin induced by BK and activation of the PLC/PKCδ/NF-κB axis [24]. In cervical tumor tissues, the B2 receptor is overexpressed compared with normal samples, as is the case in cell lines from cervical cancer. The activation of the B2 receptor was found to increase proliferation, migration, and invasion in these cells via a mechanism that involved the activation of STAT3 [15]. In gastric and hepatic cancer cells, the mechanism included activation of the ERK1/2 pathway [14]. Conversely, bradykinin via B2R was also found to enhance the migration of prostate cancer cells, in this case by activating the PKCδ, c-Src, and NF-κB pathways [13]. All these findings demonstrate the participation of kinin receptors in migration in several tumor cells. 

The preceding results have exposed that LDBK, through B1R, provokes the release of MMP2 and MMP9 in breast cancer cells, which are key molecules for extracellular matrix degradation and invasion [25]. In hepatocellular carcinoma cells, BK, via B2R, increases MMP2 secretion through the Src pathway [26,27]. BK also causes MMP2 secretion through the ERK1/2 pathway in gastric cancer cells [14]. In contrast, LDBK, through the B1 receptor, up-regulates kallikrein-related peptidases, KLK11, and KLK6, which are also related to proliferation and invasiveness [22]. These findings explain the fact that LDBK and BK increase the invasive capacity of T47D, MCF-7, and MDA-MB-231 cells. Our group, for the first time, demonstrated the greatly underexplored biological role of the B2 receptor in breast cancer. The effects induced by BK on breast cancer cells were abolished by using the B2 receptor antagonist HOE-140. In agreement with this, our results showed that BK via B2R enhances migration and invasion in T47D, MCF-7, and MDA-MB-231 cells. Similarly, the effects were observed because of the activation of the B2 receptor in glioblastoma, prostate, gastric, and cervical cancer cells [10,13,14,15]. 

In line with the findings described by others, the mechanisms involved in the migration and invasion induced by kinins and mediated by B1 and B2 receptors include the signaling pathways of ERK1/2, STAT3, PLC, PKCδ, Src, and NF-κB, among others [10,13,14,15,18]. 

Other types of signaling, such as FAK and Src, are critical for cytoskeleton assembly and membrane extension to regulate cell movement. There is evidence that kinins phosphorylate paxillin, FAK, and Src kinases in fibroblasts, keratinocytes, and endothelial cells [28,29,30,31]. Findings related to this pathway in cancer cells after kinin activation are scarce. In this way, Liu et al. described that BK, through B1R, activated FAK, triggering IL-8 expression and migration in glioma cells [23].

According to our findings, the mechanisms involved in the migration and invasion induced by kinin receptors included the FAK–Src axis, since the effects provoked by LDBK and BK were diminished when N14 and PP2 inhibitors were added to the migrating and invading cells (Figure 6a). To contribute to unveiling these pathways, future projects should study the detection of phosphorylated FAK and Src and other signaling that modulates adhesion, migration, and invasiveness in response to LDBK and BK agonists and their dependence on kinin receptors.

Due to our results, we evaluated the effect of B1 and B2 receptor antagonism in vivo. Hence, we used a model of breast cancer through the subcutaneous inoculation of MDA-MB-231 cells in female mice. Our findings revealed that the antagonism of both receptors, separately, reduced the growth in subcutaneously inoculated MDA-MB-231 cells, demonstrated by the smaller size of the tumor compared with that produced by the control MDA-MB-231 cells on day 21. This finding confirms the antiproliferative effect of des-[Arg^9^]-Leu^8^-BK and HOE-140 that we observed in vitro counting proliferation and metabolic activity of the cells. Despite this, the survival of the mice was not influenced. 

These results are in agreement with the study findings published by da Costa et al., in which they blocked the B1 and B2 receptors, separately, in a model of colorectal liver metastases in CBA males. Blocking the B1 and B2 receptors reduced the occupancy percentage and volume of the hepatic tumor; however, only the mice treated with the antagonist B1 were statistically significant [32]. They revealed that the tumor in the mice treated with B1 antagonists involved a reduction in tumor apoptosis. It is probable that B1 receptor activation has an anti-apoptotic effect on cancer cells, which is inhibited when the B1 receptor is blocked. In addition, the authors suggested that it is possible that B1R has more proinflammatory action than B2R on cancer cells since its expression is inducible compared with the constitutive expression of the B2 receptor. 

In accordance with our findings, both receptors were found to inhibit tumor growth, and it is possible that both receptors have the same effects on breast cancer cells. To date, it is unknown whether B1 and B2 receptors improve apoptosis in breast cancer cells. Thus, the mechanisms involved in tumor growth inhibition by kinin receptors are a matter for future studies.

## 4. Materials and Methods

### 4.1. Cell Culture

All the experiments described were performed in estrogen-sensitive and less aggressive MCF-7 (obtained from ATCC HTB-22-488) and T47D (obtained from ATCC HTB-133) breast cancer cells, in addition to the more aggressive triple-negative breast cancer cell line MDA-MB-231 (obtained from ATCC HTB-26), to represent tumor heterogeneity [22,25]. The MCF-7 cell line was cultured in phenol red-free DMEM (Dulbecco’s modified Eagle’s medium) containing 10% fetal bovine serum (Capricorn Scientific GmbH, Ebsdorfergrund, Germany). The MDA-MB-231 and T47D cell lines were cultured in RPMI (Roswell Park Memorial Institute) medium containing 10% fetal bovine serum (Capricorn Scientific GmbH, Germany). All the maintenance mediums were supplemented with a mix of antibiotics containing penicillin, streptomycin, and amphotericin B (Capricorn Scientific GmbH, Germany). Cultures were maintained at 37 °C in a 5% CO_2_ atmosphere.

### 4.2. Proliferation Assay

The MDA-MB-231 cells were seeded in 96-well plates at a density of 20,000 cells per well and maintained for 24 h at 37 °C and 5% CO_2_ until reaching a confluence of 80%. Subsequently, the medium was replaced with a serum-free RPMI medium to synchronize the cell cycle. After 24 h, to stop proliferation, the cells were incubated for 1 h with 25 µg/mL of mitomycin C. The effects of the peptides, 10 nM of LDBK (#3225, Tocris Bioscience, Bristol, UK) or BK (#3004, Tocris Bioscience, Bristol, UK), and overproliferation were evaluated for 24 h.

To evaluate the effect of the antagonists des-[Arg^9^]-Leu^8^-BK (#B6769, Sigma-Aldrich, St. Louis, MO, USA; AntB1) and HOE-140 (#3014, Tocris Bioscience, Bristol, UK; AntB2) on B1R and B2R, respectively, 1 µM of these compounds was used to treat MDA-MB-231 cells for periods of 48 and 96 h. Here, the cells were also treated with combinations of LDBK (10 nM) and des-[Arg^9^]-Leu^8^-BK (1 µM), or BK (10 nM) and HOE-140 (1 µM) for the same period.

Cell proliferation was evaluated using the MTT assay (Cell Proliferation Assay Kit, Item No. 10009365, Cayman Chemical, Ann Arbor, MI, USA), adding 10 μL of MTT reagent to each well and incubating the samples for 4 h (at 37 °C) so the cells began to generate formazan crystals. Next, to dissolve the formed crystals, 100 μL of the crystal-dissolving solution was added, and the samples were incubated for another 18 h. Finally, the ability of the cells to reduce tetrazolium MTT to formazan was measured at an absorbance of 570 nm in an Infinite M200pro Tecan microplate reader (Tecan Austria GmbH, Grödig, Austria).

### 4.3. Wound Closure Assay

The cells were seeded in 24-well plates for 18 h at 70–80% confluency. On the formation of a subconfluently monolayer, wounds were created with a sterile pipette tip. After wounding, detached cells were washed twice with phosphate-buffer saline, and the medium was replaced with serum-free DMEM medium to synchronize the cell cycle. The cells were then stimulated with 10 nM of LDBK (#3225, Tocris Bioscience, Bristol, UK) or BK (#3004, Tocris Bioscience, Bristol, UK) for 24 h to activate B1R or B2R. To test the dose-dependent effect of LDBK or BK, the cells were incubated at different concentrations (1–10–100 nM) for 24 h. Owing to kinin peptides inducing proliferation in cancer cells 141510, 25 µg/mL of mitomycin C (#SC-3514A, Santacruz Biotechnology, Santa Cruz, CA, USA) was added 2 h before scratching. 

To evaluate the effect of B1R and B2R antagonism, 1 µM of des-[Arg^9^]-Leu^8^-BK (#B6769, Sigma-Aldrich, St. Louis, MO, USA) or HOE-140 (#3014, Tocris Bioscience, Bristol, UK) was added 30 min before the agonists were applied.

Wound closures were monitored using a phase-contrast microscope (AE2000, MOTIC, TX, USA), and pictures were captured with 10×/0.25 Ph1 objective using a Moticam 3.0MP camera. Images were analyzed for void areas using Fiji software 2.7.0 [33]. The area of the wound was measured at time zero and at 24 h, and the difference is expressed as a wound closure percentage. 

### 4.4. Transwell Assay

Migration assays were performed using a 6.5 mm transwell with an 8.0 µm pore polycarbonate membrane insert (#3422, CORNING Life Sciences, Corning, NY, USA). The inserts were previously covered with 2 µg/mL fibronectin (#1918-FN, R&D Systems Inc., Minneapolis, MN, USA) overnight. Next, MCF-7, T47D, or MDA-MB-231 cells were seeded at 1 × 10^5^ cells per insert at the same time with agonists or antagonists, as was appropriate. The cells were allowed to migrate for 24 h to 37 °C. The cells were then fixed and stained with crystal violet (0.1% crystal violet in distilled water plus 2% ethanol) for 2 min. Using a grid eyepiece, the cells were counted in at least five fields of view at the bottom of the inserts, and pictures were captured with a Moticam 3.0 MP camera. An objective of LWD PH40×/0.60 magnification from an optical inverted microscope (AE2000, MOTIC, Schertz, TX, USA) was used. To analyze whether the effects of the B1 and B2 receptors were dependent on FAK and Src, inhibitors for these kinases were used. To inhibit FAK, 1 µM of 1,2,4,5-benzenetetraamine tetrahydrochloride (#SC-203950, Santacruz Biotechnology, Santa Cruz, CA, USA) was added, and to inhibit Src, 1 µM of PP2 (#1407, Tocris Bioscience, Bristol, UK) was added, at the same time that the agonists were applied.

### 4.5. Matrigel Assay

MCF-7, T47D, and MDA-MB-231 cells were seeded in a 6.5 mm diameter transwell covered with Matrigel (#354480, Corning Inc.). The Matrigel inserts were previously incubated with serum-free medium for 2 h at 37 °C in a CO_2_ incubator. The cells were then seeded and stimulated with agonists and antagonists as applicable. Next, the ability of the cells to invade for 24 h was evaluated. The cells that crossed the Matrigel were fixed and stained with crystal violet (0.1% crystal violet in distilled water plus 2% ethanol) for 2 min. Using a grid eyepiece, the cells were counted in at least five fields of view at the bottom of the inserts. Pictures were captured with an LWD PH40x/0.60 objective using a Moticam 3.0MP camera from an optical inverted microscope (AE2000, MOTIC, TX, USA). In addition, if the effects of B1 and B2 receptors were dependent on FAK and Src, inhibitors for both kinases were used (with the same protocol as that utilized in the transwell assay). We used the protocol as the transwell assay was applied.

### 4.6. Animal Model

Female BALBc NOD/SCID mice from the Central Animal Facility of the Universidad del Desarrollo were used. The mice were maintained in acclimatized rooms at a temperature of 22 °C in individual boxes and fed with sterile food (PicoLab^®^ Mouse Diet 20, LabDiet, St Louis, MO, USA) and water ad libitum. Mice aged nine to twelve weeks were inoculated subcutaneously with 250 µL of MDA-MB-231 cells treated or not with B1 or B2 antagonists. A total of 18 animals were used. 

Two million cells of each condition were resuspended in 250 µL of saline solution. MDA-MB-231 cells without antagonists were used as a control (Control). The animals were monitored for 21 days, and during this time, tumor size was measured with a manual caliper until the end of the experiment. The animals were euthanized on the 21st day. All experimental procedures were approved by the Institutional Committee for the Care and Use of Laboratory Animals (CICUAL), code PIA_# CMR*-2021-05.

### 4.7. Statistical Analysis

Data are presented as the mean ± standard error. A *t*-test and a two-tailed ANOVA test were used when indicated in each analysis. For the ANOVA tests, Tukey’s post-test was used. We adopted a value of *p* < 0.05 to consider the results obtained as significant. We used the Prism GraphPad software version 10 to perform all the analyses.

## 5. Conclusions

Our study demonstrates that the nonapeptides LDBK and BK are involved in the proliferation of highly metastatic cells via the activation of B1 and B2 receptors. The kinin antagonists des-[Arg^9^]-Leu^8^-BK (AntB1) and HOE-140 (AntB2) inhibit MDA-MB-231-induced subcutaneous tumor growth in vivo, demonstrating the antiproliferative role of these compounds and the future possibility of using them to pharmacologically block the effect of these receptors for the control of this disease.

Kinins, through B1 and B2 receptors, also improve the migration and invasion capacity of breast cancer cells, including estrogen-sensitive cells and triple-negative cells. 

Moreover, these processes are mediated by the FAK and Src signaling pathways. Therefore, kinin receptors and their agonists are potential targets for anti-cancer therapy. To this end, our work investigates the molecular mechanisms that regulate these processes. 

Future steps will focus on further elucidating the molecular pathways involved in the FAK–Src signaling cascade and exploring other potential signaling molecules that may contribute to the observed effects.

## Data Availability

The original contributions presented in this study are included within the article. Further inquiries can be directed to the corresponding author.

## Supplementary Materials

The following supporting information can be downloaded at: https://www.mdpi.com/article/10.3390/ijms252111709/s1.
