# Supplementary material for: Kinin Receptors B1 and B2 Mediate Breast Cancer Cell Migration and Invasion by Activating the FAK-Src Axis
Source: Int J Mol Sci. 2024 Oct 31;25(21):11709. doi: 10.3390/ijms252111709 (PMC11546324; doi:10.3390/ijms252111709)
Supplement: Supplementary file 1 [file ijms-25-11709-s001.zip › ijms-3228023-supplementary.pdf]

Kinin Receptors B1 and B2 Mediate Breast Cancer Cell Migration and Invasion by Activating the FAK-Src Axis

Felipe González-Turén [1], Lorena Lobos-González [2,3], Alexander Riquelme-Herrera [4], Andrés Ibacache [5], Luis Meza Ulloa [6], Alexandra Droguett [6], Camila Alveal [6], Bastián Carrillo [6], Javiera Gutiérrez[6], Pamela Ehrenfeld [7,8] and Areli Cárdenas-Oyarzo [9,*]

Author affiliation

[1] School of Nursing, Faculty of Health Sciences, Universidad Bernardo O´Higgins, Santiago, Chile; fegonzalez@pregrado.ubo.cl

[2] Laboratory of Cellular Communication, Program of Cell and Molecular Biology, Biomedical Sciences Institute (ICBM), Faculty of Medicine, Universidad de Chile, Santiago, Chile; lorotae@uchile.cl

[3] Center for Regenerative Medicine, Institute for Sciences and Innovation in Medicine, Facultad de Medicina, Clínica Alemana Universidad del Desarrollo, Santiago 7610658, Chile

[4] Centro Integrativo de Biología y Química Aplicada (CIBQA), Faculty of Health Sciences, Universidad Bernardo O´Higgins, Santiago, Chile; alexanderriq@postgrado.ubo.cl

[5] Laboratory of Molecular Design, Faculty of Biological Sciences, Pontificia Universidad Católica de Chile, Santiago, Chile; andres.ibacache.chia@gmail.com

[6] School of Medical Technology, Faculty of Health Sciences, Universidad Bernardo O´Higgins, Santiago, Chile; mezaulloaluis@gmail.com (L.M.); ale.droguett.z@gmail.com (A.D.); camii.alveal@gmail.com (C.A.); carrilloleonbastian@gmail.com (B.C.); tmjavieragutierrez@gmail.com (J.G.)

[7] Laboratory of Cellular Pathology, Institute of Anatomy, Histology & Pathology, Faculty of Medicine, Universidad Austral de Chile, Valdivia, Chile; ingridehrenfeld@uach.cl

[8] Center for Interdisciplinary Studies on the Nervous System (CISNe), Universidad Austral de Chile, Valdivia, Chile

[9] School of Obstetrics and Puericulture, Faculty of Medical Sciences, Universidad Bernardo O´Higgins, Santiago, Chile

*Corresponding authors, areli.cardenas@ubo.cl

Supplementary Figures

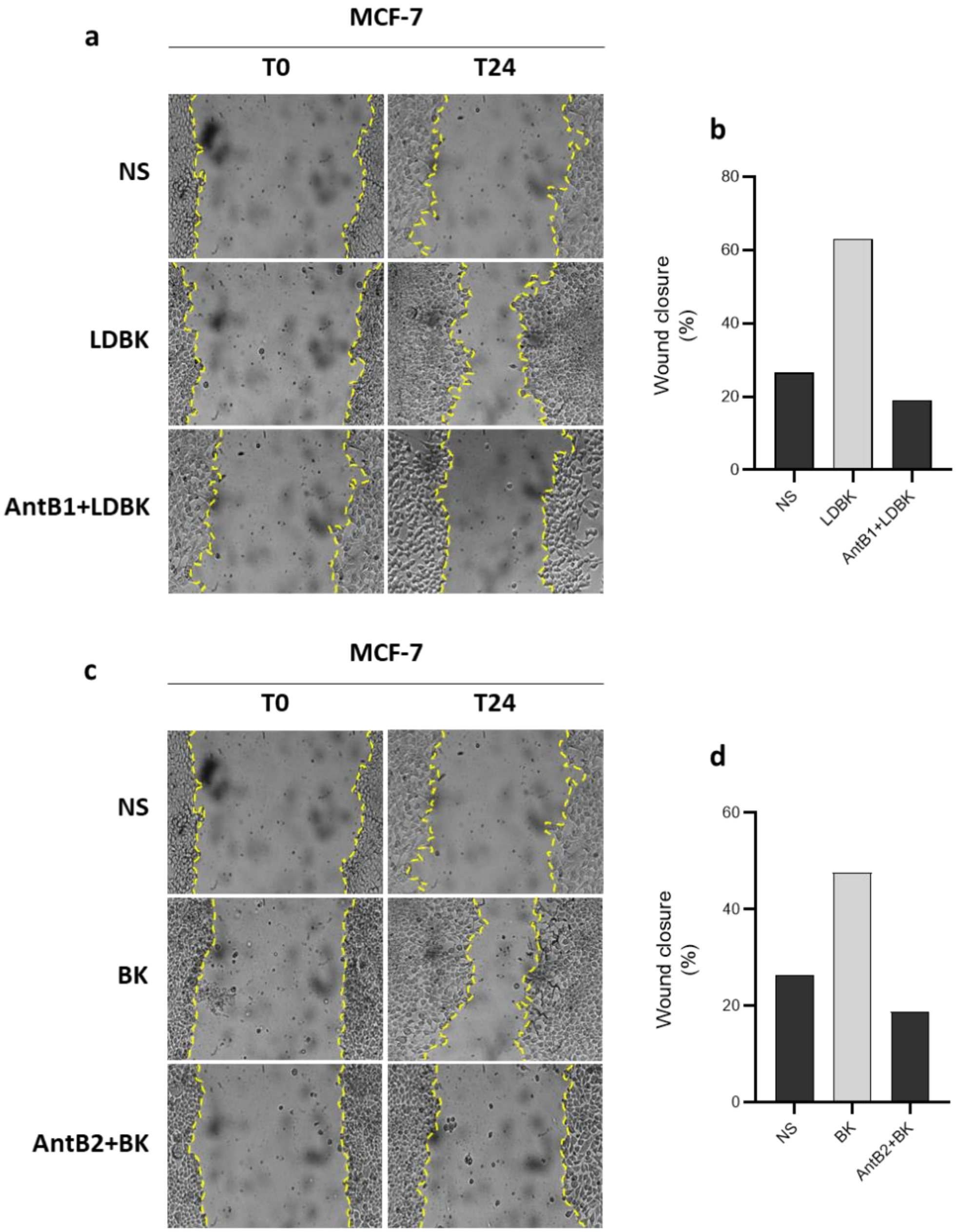

Figure S1. LDBK and BK increase the wound closure in MCF-7 cells. (a, c): scratch assay in MCF-7 cells treated with 10 nM of LDBK or BK and 1 uM of their antagonists, AntB1 or B2 AntB2, respectively. (b, d): Graph illustrates the closure percentage after 24 hours of culture, compared to non-stimulated cells (NS). Results show the mean of 5-10 pictures of one experiment.

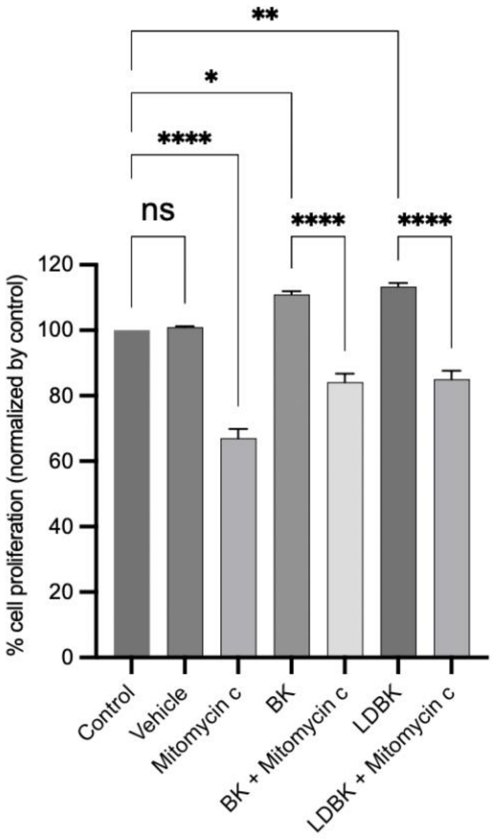

Figure S2. Effect of kinins on proliferation of MDA-MB-231 cells by Trypan Blue dye exclusion test. MDA-MB-231 cells were stimulated with 10 nM of LDBK or BK for 24 hours. Cells were previously incubated with Mitomycin C to synchronize cell cycle. Results show the mean ± SEM of three independent experiments. *p < 0.05, **p < 0.01, and ****p < 0.0001. An ANOVA two-tailed test was used.

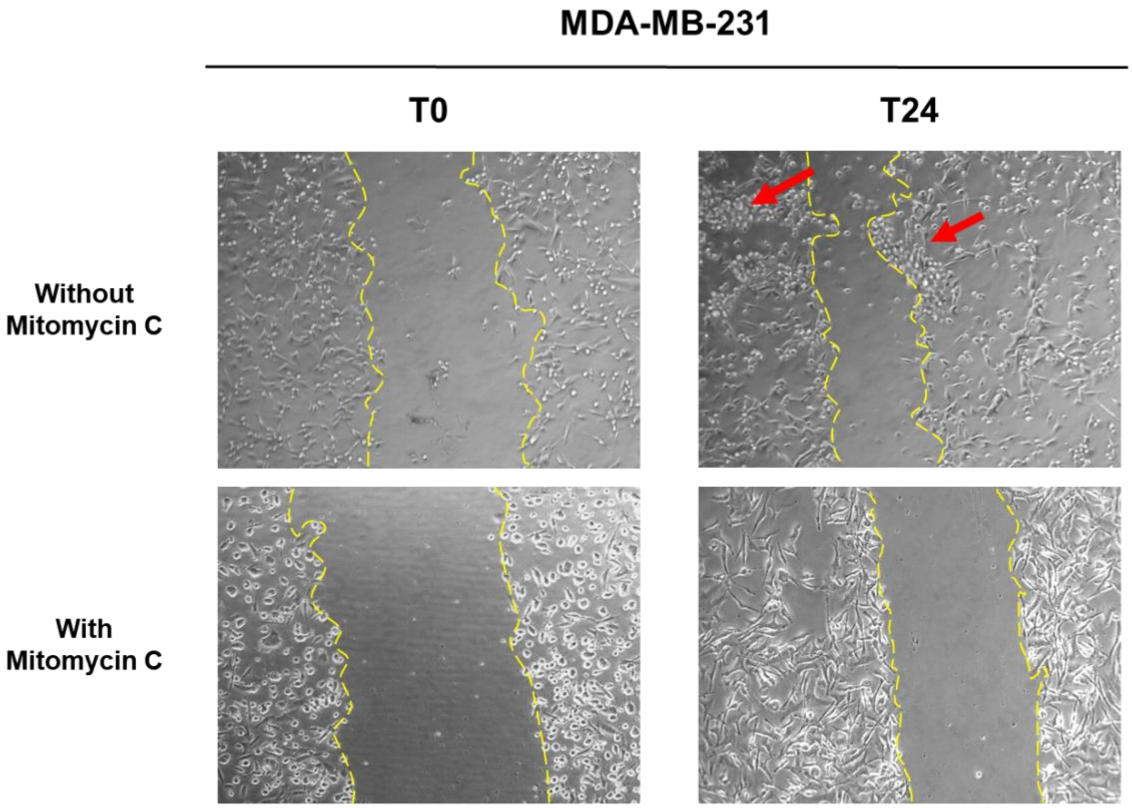

Figure S3. Mitomycin C effect on the migration of the MDA-MDA-231 breast cancer cells. The cells were pretreated or not with mitomycin C for 2 hours before scratch to inhibit proliferation. The wound was created to study migration in a cell-free area for 24 hours (T24). The red arrow indicates clusters of proliferating cells.

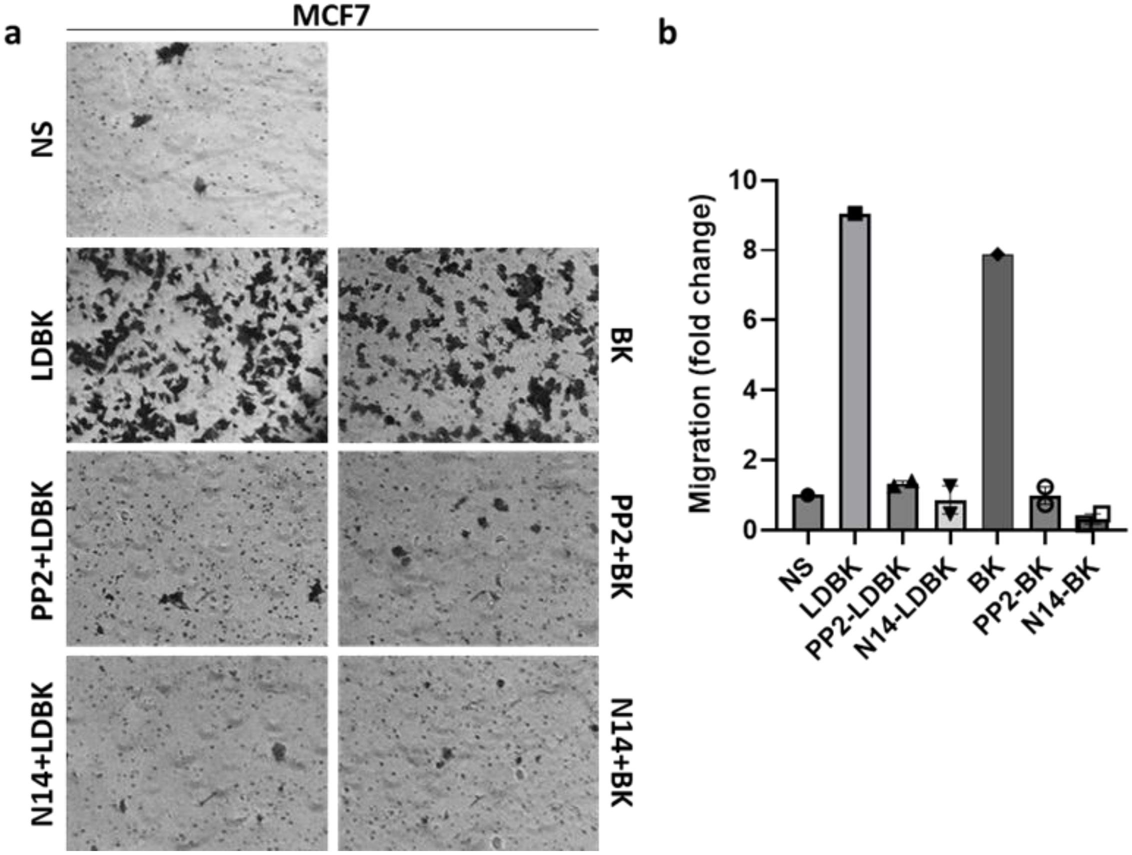

Figure S4. Kinins nonapeptides enhance migration in MCF7 cells by a Src and FAK dependent mechanism. (a): Transwell assay of MCF7 cells treated with Src inhibitor, PP2, and FAK inhibitor, N14, plus 10 nM LDBK or BK for 24 h. Cells without stimulus were considered as controls (NS). (b): The graph shows the change of migration of MCF7 cells compared to the non-stimulated cells. The number of migrating cells obtained in each condition was normalized by the control cells. Results show the mean of two independent experiments (5-10 pictures of each assay)**.**

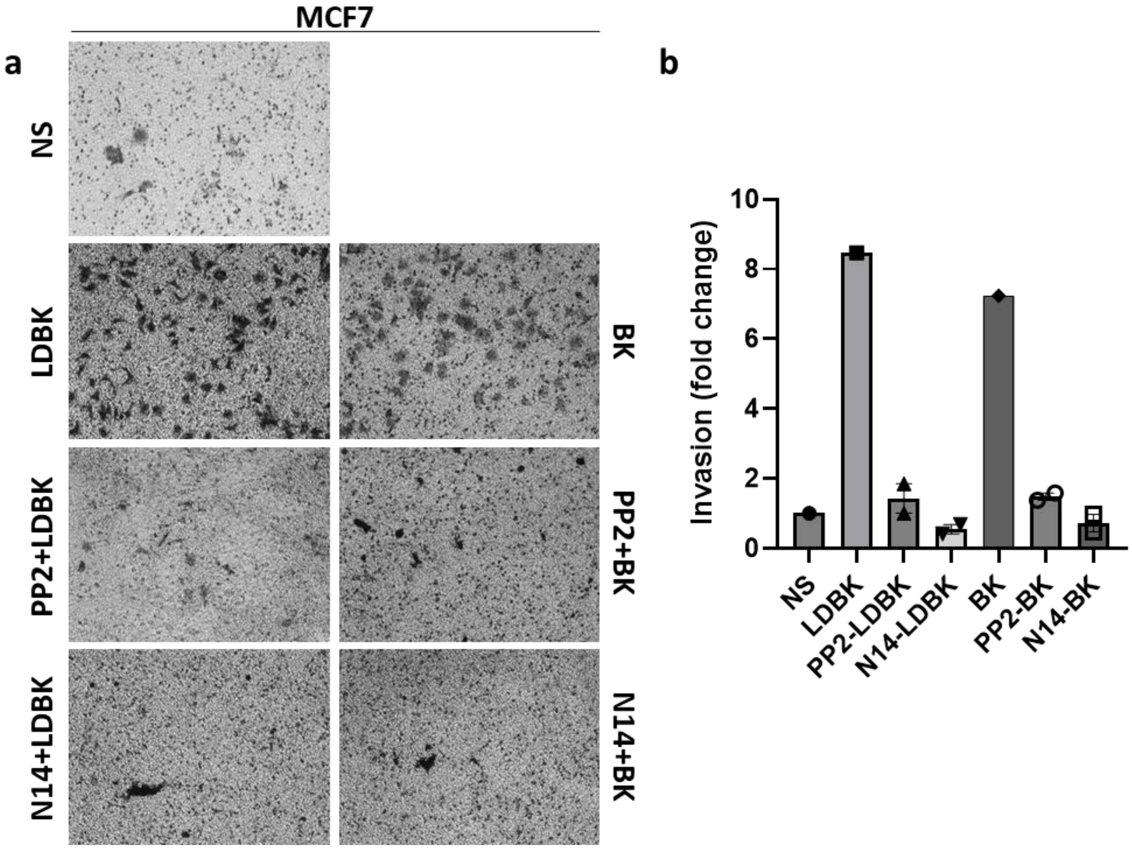

Figure S5. Kinins nonapeptides enhance invasion in MCF7 cells by a Src and FAK dependent mechanism. (a): Matrigel assay of MCF7 cells incubated with 1 uM of PP2 or N14 inhibitors plus 10 nM LDBK or BK for 24 h. Cells without stimulus were considered as controls (NS). (b): The graph shows the fold change in the stimulated MCF7 invasion cells compared to the non-stimulated cells. The number of invasive cells obtained in each condition was normalized by the control cells. Results show the mean of two independent experiments (5-10 pictures of each assay).
